# Group eye movement desensitization and reprocessing (EMDR) in chronic pain patients

**DOI:** 10.3389/fpsyg.2024.1264807

**Published:** 2024-02-22

**Authors:** Stephanie Vock, Anna Delker, Janna Rinderknecht, Felicitas Engel, Sebastian Wieland, Eva Beiner, Hans-Christoph Friederich, Ignacio Nacho Jarero, Günter H. Seidler, Jonas Tesarz

**Affiliations:** ^1^Department of General Internal Medicine and Psychosomatics, University Hospital Heidelberg, Heidelberg University, Heidelberg, Germany; ^2^Department of EMDR Mexico Research, Mexico City, Mexico; ^3^Psychotraumatologie Praxis Heidelberg, Heidelberg, Germany

**Keywords:** eye movement desensitization reprocessing, EMDR, group therapy, pain, outpatient therapy

## Abstract

The prevalence of chronic pain is increasing, and conventional pain therapies often have limited efficacy in individuals with high levels of psychological distress and a history of trauma. In this context, the use of Eye Movement Desensitization and Reprocessing (EMDR), an evidence-based psychotherapy approach for the treatment of posttraumatic stress disorder, is becoming increasingly important. EMDR shows promising results, particularly for patients with pain and high levels of emotional distress. Although group therapy is becoming increasingly popular in pain management, EMDR has mainly been studied as an individual treatment. However, a systematic review suggests that group therapy can be an effective tool for improving mental health outcomes, especially when trauma is addressed together. Based on these findings, an outpatient EMDR group program was developed for patients with chronic pain. The program consists of a total of four treatment days with 5–5.5 h therapy sessions each day and provides patients with a supportive environment in which they can learn effective pain management strategies and interact with other patients with similar experiences. Initial pilot evaluations indicate high efficacy and adequate safety for patients with chronic pain.

## 1 Introduction

Millions of people around the world suffer from pain, which has emerged as a major health problem. As populations age and chronic diseases become more prevalent, the incidence of pain is increasing, contributing to a growing societal burden (Wettstein and Tesarz, 2023). The impact of pain is not limited to physical discomfort, but also has significant social, economic and psychological consequences (GBD Disease and Injury Incidence and Prevalence Collaborators, 2017; Tesarz et al., 2019a).

Consequently, the treatment and management of pain is becoming an important part of public health. It is important to distinguish between acute and chronic pain. Acute pain is often self-limited and typically resolves as the body heals. In contrast, chronic pain, defined as pain that lasts longer than 3 months or recurs frequently, is associated with severe emotional distress and functional impairment (Treede et al., 2019). This longer duration and recurrence pattern of chronic pain underscores the complexity and severity of its impact on individuals.

Addressing chronic pain effectively remains a major challenge, as it often persists despite appropriate treatment of the underlying conditions (Buchbinder et al., 2018). Drug and invasive therapies often have little effect (Williams et al., 2020). These therapies also carry the risk of severe side effects. Opioids in particular have been shown to be harmful (Harper et al., 2021). Non-drug treatments, such as intensified psychotherapeutic treatments, are therefore becoming increasingly important, especially in the comorbid presence of mental disorders and emotional stress (Williams et al., 2020).

Psychological factors such as depression, anxiety, the lack of self-efficacy and catastrophizing, collectively referred to as negative effects, are often already indications that pain is going to persist. Catastrophizing in particular, a pain-related construct characterized by negative cognitions, helplessness, pessimism, rumination and the fact that patients significantly overestimate pain-related symptoms, is a variable that appears to have a decisive influence on pain chronification. At the same time, these negative affects are also consequences of the constant confrontation and the experience of permanent and recurring pain (Edwards et al., 2016). Other consequences of chronic pain are fear of illness and somatization, lower self-esteem and lower self-efficacy. Furthermore, chronic pain patients report a stronger impairment of their emotional functionality, often also referred to as distress (Burke et al., 2015). Since psychological factors play a decisive role in chronic pain, both causally and supportively, psychotherapeutic methods have proven to be helpful.

However, the range of pain psychology therapies is limited and therapists often lack pain-specific training (Darnall et al., 2016). Group therapy can be a promising approach as it allows more patients to be seen, while creating a supportive environment where patients can learn effective pain management strategies and share experiences with others who have had similar experiences.

Although psychological therapies can improve the quality of life and functioning of people with pain, their impact on the alleviation of pain is often limited (Williams et al., 2020). In particular, patients with severe comorbid mental illness and a history of trauma pose a significant challenge to clinicians, as many psychological pain therapies do not adequately address trauma and posttraumatic stress symptoms (Lumley et al., 2022). Unfortunately, these patients are often excluded from group opportunities because of the fear that they will overwhelm the group or be re-traumatized. This exclusionary approach is inadequate, given that psychological distress is frequently observed in patients experiencing pain (Afari et al., 2014; Fishbain et al., 2017; Kind and Otis, 2019). This results in a significant number of patients missing out on potentially beneficial treatments. Fortunately, there exists a suite of successful strategies to tackle psychological trauma that are easy to learn and apply.

Numerous studies have confirmed the link between psychological trauma, PTSD, and chronic pain, with reported prevalence ranging from 24% to over 80% in various studies. In a review by Liedl and Knaevelsrud (2008), the connection between chronic pain and trauma-related disorders is made clear using various models.

The “mutual maintenance model” proposed by Sharp and Harvey suggests an interdependent relationship, where pain and PTSD symptoms interact and perpetuate each other. The model includes seven mechanisms, for example avoidance, memories of the trauma, but also fear and pain perception. An alternative explanatory framework is the “shared vulnerability model” proposed by Asmundson et al. This model suggests a predisposition—potentially even genetically rooted— that contributes to the onset of both PTSD and chronic pain. Individuals who experience both chronic pain and PTSD have an increased sensitivity to anxiety. This sensitivity involves heightened individual reactivity and exaggerated catastrophic responses to physical indicators of heightened anxiety. Previous models have tended to focus on individual facets of the relationship between pain and trauma. However, Norton and Asmundson have extended the extensively researched “fear-avoidance model” to include physiological arousal. This extension reveals a positive feedback loop in which physiological elements intersect with cognitive and behavioral aspects, hindering the adoption of effective coping mechanisms. Increased overall physiological arousal increases both the actual experience of pain and the belief that activities will increase pain (Sharp and Harvey, 2001; Asmundson et al., 2002). The complex and multifaceted interaction between trauma, PTSD and pain has led to the early discussion of the use of Eye Movement Desensitization and Reprocessing (EMDR) in the treatment of patients with pain and trauma.

EMDR is an evidence-based psychotherapy approach for the treatment of psychological trauma that is easy for therapists to learn and apply (Laliotis et al., 2021). EMDR involves exposure, dual focus of attention and bilateral stimulation to process traumatic memory and reduce associated distress (Laliotis et al., 2021). Unlike conventional Cognitive Behavioral Therapy (CBT) approaches, which are usually based on classical learning theories, EMDR is based on the Adaptive Information Processing (AIP) model. This model posits that the brain naturally seeks psychological balance, similar to the body’s physical healing processes. According to AIP, mental disorders and illnesses arise from memories of traumatic events that are not fully processed or integrated into the brain’s neural memory networks. These unprocessed, dysfunctionally stored memories can be reactivated by certain stimuli, leading to psychopathological symptoms such as PTSD, negative effects, and bodily symptoms. Whereas classical exposure methods in CBT involve systematic and controlled confrontation of the patient with anxiety-provoking stimuli or situations, such as detailed descriptions of events or direct questioning of beliefs, in order to reduce anxiety responses and develop coping strategies, the exposure principle of EMDR uses synchronized eye movements in conjunction with a dual focus of attention. This approach facilitates the incorporation of maladaptively stored memories into the brain’s adaptive neural networks. This process alleviates psychological distress and enhances adaptive responses to future stimuli, thereby promoting psychological resilience and health (World Health Organization, 2013).

EMDR is recommended in clinical practice guidelines as a first-line treatment for PTSD (Martin et al., 2021). Due to its proven effectiveness in treating psychological trauma, EMDR is now also recommended by World Health Organization (2013). Given the high comorbidity of chronic pain and psychological trauma, and EMDR’s ability to address body sensations and experiences, it is not surprising that EMDR is increasingly being used in the treatment of chronic pain (Matthijssen et al., 2020).

Several randomized controlled trials have demonstrated the efficacy of EMDR in the treatment of chronic pain conditions such as chronic musculoskeletal pain (Gerhardt et al., 2016), back pain (Gerhardt et al., 2016), headaches (Konuk et al., 2011), phantom limb pain (Rostaminejad et al., 2017), fibromyalgia (Friedberg, 2004), and rheumatoid arthritis (Tesarz et al., 2019b; Matthijssen et al., 2020). These studies have demonstrated the effectiveness of EMDR in reducing pain intensity, pain-related disability and associated psychological distress (Leisner et al., 2014). However, most of these studies examined EMDR as an individual treatment rather than in a group format, which is surprising given that group interventions are commonly used for both chronic pain and PTSD (Matthijssen et al., 2020). Given the benefits of group therapy, it is important to further explore the potential of EMDR group therapy as an effective treatment approach for patients with chronic pain and comorbid psychological trauma.

While individual EMDR therapy has been shown to be effective in treating psychological trauma (National Institute for Health and Care Excellence, 2018), interest in the effectiveness of group EMDR has increased in recent years (Kaptan et al., 2021). Group EMDR therapy has been shown to be particularly effective when large numbers of people are affected by the same event, such as after major events and natural disasters (Jarero et al., 2008). A 2021 systematic review indicates that the group format can be an effective tool for improving various mental health outcomes, including PTSD, depression and anxiety (Kaptan et al., 2021). The group EMDR approach is particularly appropriate when there is collective trauma, such as after earthquakes or tsunami disasters. In such situations, group therapy can create a supportive and empowering environment where participants can share their experiences and learn coping strategies from each other (Jarero et al., 2006).

This observation leads to the consideration of using group EMDR therapy in the treatment of chronic pain. Pain can be seen as a form of traumatic experience, and coping with the pain trauma together in a group therapy setting can be an effective therapeutic step.

The effectiveness of EMDR group therapy has been demonstrated in numerous studies (Kaptan et al., 2021). In addition to EMDR-specific factors in the sense of the AIP model, a number of other non-specific factors can be considered as underlying effective factors in the group setting. Yalom, for example, identified seven general factors of influence, such as the therapeutic relationship, group cohesion, interactions between members, the universality of suffering and the importance of the group process for individual change (Yalom, 2005). Accordingly, the shared experience of pain can help create a cohesion and understanding among group members, and this supportive environment can facilitate the use of EMDR-derived techniques. In addition, EMDR therapy can help patients to process the traumatic aspects of their pain experience, which may help to reduce pain intensity and associated psychological distress. This could help promote adaptive coping and alleviate pain.

Given the high comorbidity of chronic pain and psychological trauma and the proven efficacy of EMDR therapy in treating both conditions, an innovative outpatient EMDR therapeutic group program was developed for patients with chronic pain. The program aimed to provide a supportive and empowering environment in which patients could learn and practice effective pain management strategies, process traumatic memory and make supportive connections with other group members having similar experiences. In this article, we would like to introduce the treatment program and report on our initial experiences with its use, including its feasibility and acceptability among patients with chronic pain. We hope to contribute to the growing body of evidence supporting the use of EMDR therapy in the treatment of chronic pain and psychological trauma through a comprehensive evaluation of this EMDR group program.

### 1.1 Current scientific evidence on EMDR group therapy

The effectiveness of EMDR therapy used in groups to treat mental disorders in adults and children was examined in a recent review (Kaptan et al., 2021). The review identified 22 studies with a total of 1,739 participants that examined four different EMDR protocols specifically designed for group therapy. These protocols adapted the eight standard phases of EMDR therapy to group therapy. They were: (1) the EMDR Integrative Group Treatment Protocol (IGTP) (Jarero et al., 2008), (2) the EMDR Integrative Group Treatment Protocol for Ongoing Traumatic Stress (EMDR-IGTP-OTS) (Jarero et al., 2006, 2018), (3) the Group Traumatic Episode Protocol (Lehnung et al., 2019; Korkmazlar et al., 2020), and (4) the EMDR Group Child Protocol (Korkmazlar et al., 2020). The EMDR IGTP protocol had the strongest evidence base with 13 studies, followed by EMDR IGTP OTS with four studies and G-TEP with four studies. Only one study was available for the EMDR-GP/C protocol. Despite the heterogeneity of the studies in terms of sample, setting, outcomes and number of sessions, EMDR group interventions significantly reduced post-traumatic stress, depression and anxiety symptoms after treatment compared to pre-treatment or control groups. However, the methodology of the studies was subject to a relevant risk of bias, suggesting that further studies with sound methodology, larger samples and sufficiently long follow-up periods are needed to determine the effectiveness of EMDR group therapy. Furthermore, no studies of EMDR group interventions for chronic pain were identified in this comprehensive systematic literature review, highlighting the importance of our work.

## 2 Methods

The present study aimed to develop an intensified EMDR therapy group program for patients with chronic pain. To achieve this goal, a series of expert interviews and workshops were conducted with therapists experienced in running EMDR therapy groups. In addition, two focus groups with seasoned therapists experienced in delivering EMDR to patients with chronic pain were included, complemented by the participation of therapists and physicians experienced in pain management and two EMDRIA certified EMDR consultants. These interviews and workshops provided insights and recommendations relevant to the development of the enhanced EMDR group treatment protocol. Two pilot groups were conducted to test the feasibility and acceptability of the protocol, the results of which ultimately contributed to the development of the enhanced EMDR group treatment protocol. Patients’ experiences and changes in symptoms during the intervention were recorded descriptively and exploratively in an open feedback session following the intervention as well as in an anonymous online survey with the “Patient Global Impression of Change” (PGIC) scale (Guy, 1976; Dworkin et al., 2008) and on possible side effects 1–2 weeks after the intervention. As this was a development phase in which the intervention program was to be adapted and improved, the survey focused on participants’ open feedback on the content, global impression of change, safety and possible side effects. The evaluation asked about personal impressions, the most and least helpful elements of the therapy as well as individual suggestions for improvement, and negative aversive events during and after the therapy. Based on these data, we developed the intensified EMDR group treatment program for the treatment of chronic pain, presented here.

## 3 Contents of the intensified EMDR group program

Our final intensified EMDR group program extends over four treatment days of 5–5.5 h each day (see Table 1). It is based on three pillars: (1) EMDR-based exposure and resource work, (2) education, and (3) physical activation. The education prepares patients for the actual exposure work, strengthens patients’ belief in the therapy and is intended to help patients engage in the EMDR approach and physical activation as well as strengthen accompanying adaptive coping behaviors.

**Table 1:** Treatment program overview.

Preliminary interviews	Day 1 1:00 p.m. to 6:30 p.m.	Day 2 1:00 p.m. to 6:00 p.m.	Day 3 1:00 p.m. to 6:00 p.m.	Day 4 1:00 p.m. to 6:00 p.m.
Phone screening	Welcome Getting to know each other	Arrival	Arrival	Arrival
Individual preliminary interviews	Education I (Pain)			
	Education II (EMDR and protocols) PT-OTS pain episodes	G-TEP-pain episodes	G-TEP-pain episodes	G-TEP-pain episodes
	Active walking	Active walking	Active walking	Active walking
	PT-OTS	PT-OTS- Pain episodes	PT-OTS-pain visualization	Pain absorption exercise
	Short physical activation	Short physical activation	Short physical activation	
	Break	Break	Break	Break
	Conclusion	Education III (pain and pain memory)	Education IV (pain and stress)	Conclusion and feedback

IGTP-OTS, EMDR Integrative Group Treatment Protocol for Ongoing Traumatic Stress; G-TEP, Group Traumatic Episode Protocol.

The combination of psychoeducation and physical activation is based on a study by Van Woudenberg et al. (2018), which showed that this is an effective and efficient approach in the context of intensified exposure programs. This is also supported by the work of Liedl and Knaevelsrud (2008), who emphasizes the positive effects of physical activity. The use of education is a central component of many guidelines, particularly in pain management. Recent studies also show that reinterpreting pain as a neurobiological correlate (rather than a physical defect) is therapeutically effective (Ashar et al, 2022, 2023). In addition, physical activity was chosen as a complementary component because some of the participants were very exhausted by the therapy and physical activity could have a positive and complementary counteracting effect. In this context, we have decided to use guided walking as a physical activity and to give patients the opportunity to get to know each other better in a casual context and to process the EMDR sessions.

The program is run by a therapist together with a co-therapist, and the roles can be alternated. All participants received a telephone screening interview prior to participation in the group program, as well as a pre-interview with one of the group therapists. The therapy content is based on exposure through individual group EMDR work using adapted EMDR group protocols, complemented by resource and pain management-oriented group work, physical activation and education. This setting allows for the development of stable group cohesion so that both (1) the group therapy setting can be used to facilitate the change of unhelpful pain-related thoughts and behaviors, and to foster social support through group interactions; and (2) individual trauma work can be conducted in the group setting.

### 3.1 Therapeutic elements of the intensified EMDR group program

To optimize exposure and trauma processing work in our treatment program, we chose to integrate two different EMDR group therapy protocols: the EMDR Integrative Group Treatment Protocol for Ongoing Traumatic Stress (EMDR-IGTP-OTS) (Jarero et al., 2018) and the Group Traumatic Episode Protocol (GTEP) (Lehnung et al., 2019). Both treatment protocols were slightly modified for our program by specifically targeting distressing material related to pain (processing episodes as part of the patient’s “mental pain film”) or directly targeting the pain itself. Through these adaptations, we were able to ensure that the therapy content was tailored to the specific needs of our patients, allowing for effective processing of traumatic events and pain symptoms. This approach of individual group EMDR work to process stressful experiences and joint group work for resource activation was found to be very supportive and enriching by the patients.

#### 3.1.1 Telephone screening and preliminary individual therapy session

Before admission to the group therapy setting, a telephone screening and a preliminary individual therapy discussion took place with all participants (see Table 2). During the screening, the patients were given important information about the group, the format, the procedure, and the time frame. In addition, patients had the opportunity to clarify their own questions regarding the therapy. This procedure is of great importance as it gives patients a clear idea of what to expect and how the therapy will proceed. In addition, any concerns can be clarified in advance, which increases confidence in the therapy and can support the success of the treatment. Before the group therapy began, there was also an individual preliminary interview, which was conducted by the group therapists and lasted between 20 and 30 min. In this individual session, the therapists obtained an overview of the patient’s medical history, explored the patient’s treatment motivation, and asked about the patient’s subjective pain history, psychological comorbidities as well as important resources and competences. They were also asked about their expectations of the therapy. Information about the timing of the group and any exclusion criteria were assessed. This preliminary consultation enabled the therapists to better understand the individual needs and prerequisites of the patients and to adapt the therapy accordingly. In these discussions, information was also provided about the setting, the exposure character and the background of EMDR in trauma and pain (such as the AIP model, pain memory and the importance of dysfunctional memories in pain chronification).

**Table 2:** Content of the preliminary interviews.

Preliminary interviews	Content
	■ Individual preliminary talks of 20–30 min by the group therapists (by telephone, or therapist’s consultation) ■ Client history and motivation check ■ Subjective pain history, psychological comorbidity, important resources and competences of the patient ■ Expectations of therapy ■ Brief instruction EMDR and pain, information about side effects ■ Information on the group schedule, advice on weatherproof clothing ■ Exclusion criteria ■ If necessary, send a patient brochure on EMDR and pain

#### 3.1.2 The EMDR group protocol for ongoing stress

The EMDR Integrative Group Treatment Protocol for Ongoing Traumatic Stress (EMDR-IGTP-OTS) is an enhanced version of the EMDR Integrative Group Treatment Protocol (EMDR-IGTP) developed by Jarero and Artigas (2012). Its primary objective is to offer a therapeutic framework for individuals who do not experience a subsequent post-traumatic safety window, such as those facing persistent emotional distress and ongoing traumatic exposure, including cases related to cancer. The EMDR-IGTP-OTS aims to facilitate the reprocessing of traumatic memories in such individuals.

Compared to other protocols, the EMDR-IGTP-OTS mainly uses drawings and symbols to reprocess the distressing memories. This leads to a deceleration of the process and is usually perceived by the participants as less stressful and more controllable. The version adapted for the specific need of patients with pain specifically offers the possibility to directly address the pain and the stressful experiences associated with it. The EMDR-IGTP-OTS is simple to conduct and requires no special equipment or materials. All that is needed is blank A4 sheets of paper and crayons to draw on. There is no sharing of experiences in the group except for brief feedback after doing an initial stabilization exercise at the beginning: Strictly speaking, the IGTP-OTS is therefore not an EMDR group therapy, but a protocol for conducting (individual) EMDR therapy in a group setting. Also, the history taking is done individually and not in the group. This means that there is only a small risk of group participants being overwhelmed by the experiences of other participants. For alternating bilateral stimulation, the EMDR butterfly hug method for self-administered bilateral stimulation (Jarero and Artigas, 2023) is recommended. It involves the individual crossing their arms and alternately tapping their chest with their hands, creating a soothing rhythmic sensation that helps reduce distress and facilitate the processing of traumatic memories during EMDR sessions. This method promotes a sense of calm and safety, enhancing the effectiveness of the therapy. Overall, the protocol is based on the classic eight phases of the EMDR standard procedure.

- Phase 1: This phase includes the client’s anamnesis, which is taken individually. Accordingly, each group participant receives a detailed individual interview before the start, in which the therapist has the opportunity to learn details about the trauma history as well as important resources of the patient. This part with done beforehand within the preliminary individual therapy session before inclusion in the intensified EMDR group program.
- Phase 2: In the next phase, the therapists briefly introduce themselves and it starts with a psychoeducation session, the introduction to the self-soothing exercises, the Subjective Units of Disturbance Scale (SUDS) and the teaching of how to perform the butterfly hug.
- Phase 3: The next step is to prepare for doing the exposure work (“assessment phase”). For this, the participants divide the blank A4 sheet in front of them into four rectangles and label them A, B, C, and D. In order to capture the full spectrum of traumatic stress, participants are asked to run a mental movie of their entire pain history, from right the beginning until today, or even to look into the future. In the first session, participants are asked to choose the hardest, most painful or distressing moment *“From the whole mental movie, please choose the hardest, most painful, or distressing moment…*” Participants are instructed to observe which emotions and physical sensations accompany that memory at this moment. Then the participants draw this moment symbolically or in their own way in the square with the letter “A” and rate the corresponding disturbance level (subjective units of disturbance, SUD). Alternatively, in the pain therapy setting, the pain itself can be focused on with the help of pain visualization (“*But you can also try to shape the pain as such. The following questions help: If the pain had a colour, what would it be? If the pain had a shape, what would that be? If the pain had a different texture, what would it be? Would it be hard or soft? Rough or smooth?*”).
- Phase 4: The next step is to lead the group into reprocessing together (“desensitization phase”). For this, the participants are asked to consciously put themselves back into the moment chosen in square A, applying the bilateral stimuli in the form of butterfly hugs until they feel in their body that it has been enough (1–3 min on average). The duration of exposure and bilateral stimulation thus varies from individual to individual and is based on the participants who need the longest. When about 90% of the participants have finished the butterfly hug, it is usually possible to move on to the next step. After this desensitization procedure, the participants are asked to draw in the next square of the worksheet (B) how they feel now and to assess the corresponding disturbance level (SUD).
- These instructions for square B and the procedure are repeated for square C and D, so that a total of four times the distressing memory is reprocessed and the respective degree of disturbance is evaluated. After the reprocessing of the last square, the participants are asked to look at all the drawings and choose the one that disturb them the most. Then, participants are asked to turn the paper over and write down the corresponding SUD they feel now. Deliberate care is taken to ensure that participants do not make the mistake of copying only the SUD of the most distressing drawing but write down the SUD of the disturbance they are feeling NOW… IN THE PRESENT MOMENT.
- Phase 5: The next step focuses on the vision of the future. For this, participants are guided to draw how they see themselves in the future and title their drawing. In this phase, it is NOT necessarily about imagining a happy future/outcome or successfully coping with an expected future event, but it is more important that participants are authentic and honest with themselves than forcing positivity. It should be noted that in contrast to the standard EMDR protocol in the individual setting, the installation of a positive cognition is not provided in the context of this EMDR group protocol, since in the context of OTS work each participant may have a different point in time for reaching an ecological level of distress, and thus at the end of a session one or the other may well still have blocking beliefs that get in the way of installing a positive cognition.
- Phase 6: Participants are instructed to remember the drawing that disturb them the most, close their eyes and scan their body from head to feet and at the end to do the butterfly hug.
- Phase 7: Instead, a joint relaxation exercise is carried out with all participants at the end of the protocol (“closure phase”), in order to release as many participants as possible from the intervention in a positive state. It is recommended that the drawings and worksheets remain with the therapists. This can symbolically underline that the burden is given or left behind—and not taken home.

#### 3.1.3 The group traumatic episode protocol

The Group Traumatic Episode Protocol (G-TEP) provides a group therapy approach for individuals who have experienced life-changing and traumatic events that continue to have lasting effects. This protocol focuses on the entire traumatic episode, including the initial traumatic experience and all related events, regardless of how recent they may be. This adapted version is applicable even in the absence of a history of life-changing and traumatic events. The protocol can therefore be used with any patient in pain who reports distressing experiences associated with their pain. The therapeutic process is highly specialized, with the therapist leading participants through a step-by-step exposure to the traumatic memories while maintaining dual attentional focus using bilateral stimuli. The session is designed so that a single worksheet takes you through the process step by step. The processing of stressful events is not necessarily chronological. Usually, about 3–5 stressful events, so-called “points of distress” (=PoDs) are identified. Processing usually takes place in several (2–4) sessions of 70–90 min each, possibly on consecutive days. In contrast to the EMDR-IGTP-OTS protocol, the G-TEP protocol deliberately provides for the exchange of experiences in the group: however, the exchange of experiences among the group participants is limited to the stabilizing elements and resource activation; the conscious exchange of participants about stressful/traumatic events is limited. The treatment setting should include at least one EMDR therapist and one psychosocial professional or trained assistant for every 10 participants.

For bilateral stimulation, the participants’ self-performed tapping on their worksheet is used together with eye movements (as the participants follow their own fingers moving back and forth on the worksheet), the Butterfly Hug is only for installing resources. Overall, the protocol is based on 8 different steps through which the participants are guided step by step by the therapist.

- The protocol starts with a specific stabilization exercise called the “Four elements exercise” which is repeated at the end of each session. The exercise involves focusing on four elements—earth, air, water and fire—and identifying personal associations with each element. The exercise combines different levels of stress reduction: the element “earth” corresponds to grounding, the element “air” corresponds to the regulatory effect of breathing, the element “water” means the specific increase of saliva production to stimulate a vegetative relaxation reduction and the element “fire” refers to the imaginative power of visualizing through the installation of a safe place. By engaging with these elements, individuals can ground themselves in the present moment and develop a sense of safety and stability. The exercise can later be done independently by the participants outside the group setting.
- After activating resources, participants are asked to name the initial traumatic event they would like to work on. They can do this either through a short description or a drawing on their worksheet and then assess the corresponding SUD.
- Before the actual trauma exposure begins, a past and a future resource are first activated. Participants are asked to recall a positive memory and draw or describe it on their worksheet. This is then anchored by the butterfly hug.
- To activate a future resource, participants choose a positive cognition to represent how they would like to think about themselves in the future. This is also recorded on the worksheet to support meta-communication. The trauma is presented on the worksheet surrounded by current, past and future resources to illustrate that the event is in the past and that there is safety in the here and now as well as that the future holds hope.
- In the next steps, the participants are guided into the actual process work. To do this, first the relevant targets, the PoDs are identified through a non-sequential Google search (“*Now let the whole episode, everything that happened, from the beginning until today, run in front of your inner eye, like a Google search on your computer. Look for something that is still bothering you, in no particular chronological order*”). Participants identify the PoDs of the traumatic event in no pre-given order and process them one by one, tapping from one side of the worksheet to the other.
  After identifying a PoD, participants are instructed to visually represent it on their worksheet by either drawing or writing. To initiate Bilateral Stimulation (BLS), all participants are asked to touch the “Disturbance” box (representing the past) and then the “Safe Place” box (representing the present) with one hand. They are instructed to follow their hand with their eyes while doing so, until they have identified a PoD. Once a PoD is found, participants document and enter it into the corresponding box on their worksheet. There are different options for the length of the bilateral stimulation: For example, the length of each set can be set by the group leader (e.g., 10 or 20) and done synchronously together in the group under the guidance of the group leader. The advantage of this joint tapping is that it promotes group cohesion. The disadvantage is that the perceived appropriate length of the tapping can vary individually. Alternatively, therefore, the length of each set can be left open by the group leader so that everyone can tailor it until a change is noticed during the tapping or a break becomes necessary. It should be noted that the “Eye Movement Desensitiziation”-strategy of this approach here focuses strongly on the PoD, in contrast to the classical EMDR setting, where association chains fostered. That is the dual focus of attention returns there again and again and inquires the level SUD (Subjective Unit of Disturbance), which however imposes a limit on the chains of associations. The protocol recommended to use six to nine sets of BLS to reduce the SUD for each PoD as much as possible. After each BLS set, participants are asked to pay attention to their thoughts, feelings, body sensations or whatever they perceive. After the 3rd, 6th, and 9th BLS sets, the focus is turned back to the PoD and the SUD is rated on the scale from 0 to 10 and recorded on the worksheet. These exposures and desensitization steps are carried out by each participant individually, no exchange with other group participants is planned in this phase.
- After the 9th bilateral stimulation set of a PoD, the SUD level is finally assessed. If the SUD has dropped to an ecological appropriate level, the next PoD can be identified by another “Google search” and processed according to the same procedure (3 × 3 sets á 10–20 BLS). Participants whose episodes SUD is still very high (SUD > 5), should continue with the same PoDs. It is recommended to work on a minimum of 2–3 PoDs per session.
- Thereafter, participants once again rate their SUD level for the whole episode and choose a positive cognition to anchor (“*How would you like to think about the whole episode now? What did you learn? What do you take away from today?*”). At this point, sharing their experiences in the group can be helpful. Then the cognition is written down or drawn and anchored with the butterfly hug.
- Finally, after anchoring the positive cognition, the four elements exercise is done once again. At the end of a session, the group leader should assess how each participant is feeling and identify who needs more time to work through the stressful material and offer additional support if necessary.

#### 3.1.4 The pain absorption exercise in the group

A modified version of the classical EMDR “absorption technique” (Hofmann, 2009) was used for the group setting. The aim of this modification was, on the one hand, to specifically address the patients’ pain problems and, on the other hand, to integrate them into the group setting in such a way that the advantages of the group could be used.

The basis of the modified absorption technique is the same as in the classical absorption technique and is based on the principle of linking a specific stressful situation with individual resource-rich memories and sensations. However, the focus of the modified version is on the pain. Here, the pain is first visualized and the associated degree of stress is assessed. In the next step, participants are asked to identify individual skills or competencies and the positive body feeling associated with them. These are anchored through bilateral stimulation by means of a butterfly hug. In the last step, the resources are used to address the visualized pain and reduce the associated stress level.

In total, seven steps can be distinguished in this exercise. The exercise starts with a short explanation and general preparatory instructions (step 1). Then the participants are asked to visualize their pain (step 2) and to assess the associated current stress level with the help of the SUD scale (step 3). In the next step (step 4), the participants are asked to name different skills and competences that can particularly help them to cope with their pain. This process is done as a group work in order to use the group as a supportive and empowering environment.

Subsequently (step 5), two individual competencies are selected by each participant and these are anchored together with the memory of a situation in which they succeeded in using this skill and together with the corresponding body feeling by means of bilateral provocation. At the end of the exercise, the original pain image is returned to and bilaterally provoked against the background of the two resources (step 6). Finally, the degree of stress is evaluated again (step 7). The modified absorption technique thus offers an effective way of working on individual pain problems in a group setting, drawing specifically on individual resources. The group work enables the exchange and strengthening of individual pain management strategies as well as the establishment of helpful relationships with other patients who have had similar experiences.

### 3.2 Evaluation of the group—exploratory evaluation

All patients suffered from chronic pain, mainly in the musculoskeletal system. However, we included all eligible patients regardless of pain diagnosis. Patients ranged in age from 19 to 63 years and all but one were female. Inclusion criteria for participation in the group were chronic primary or secondary pain, willingness to participate in group therapy and attendance at all scheduled appointments. In addition, patients had to be able to self-regulate sufficiently and not fulfill any of the absolute contraindications such as suicidal tendencies (see Table 3). During the qualitative follow-up survey, participants expressed significant benefits derived from two key aspects: the opportunity to connect with others who share similar experiences, and the utilization of EMDR-specific techniques. These aspects were highlighted as particularly valuable and impactful for the participants. Assessing potential side effects, no relevant side effects were reported in connection with the therapy. Furthermore, it is noteworthy that none of the participants reported a strong or very strong worsening of their symptoms during the course of therapy. These findings reinforce the notion that our therapeutic program is both safe and well tolerated within the group setting. Taken together, the evaluation reveals positive outcomes and a high level of satisfaction among participants. The positive impact of group dynamics, coupled with the effective application of EMDR-specific techniques, demonstrates the efficacy and potential of our therapeutic program for individuals experiencing chronic pain and emotional distress.

**Table 3:** Possible contraindications.

Severe dissociation or psychosis	■ Individuals with severe dissociative or psychotic symptoms may have difficulty engaging with the structured and focused nature of group therapy and may require more individualized treatment to address these symptoms.
Active suicidal ideation, severe self-harming behavior	■ People who have active suicidal or severe self-harming behavior may need more intensive and individualized treatment than can be provided in a group setting.
Insufficient social stability	■ Individuals who lack structural (social) and functional (family) support may need more intensive and individualized treatment than is possible in a group setting.
Active substance abuse/dependence	■ People who actively use or are dependent on drugs or excessive alcohol may not be suitable candidates for group therapy as they may not be able to fully engage with the therapeutic process.
Inability to tolerate a group setting	■ If severe anxiety or other problems make group participation significantly more difficult, individual therapy may be a more appropriate form of treatment.
Lack of motivation or commitment	■ Group therapy requires a certain level of motivation and commitment from the participants. If individuals are not willing or able to engage in therapy, they cannot benefit from the treatment and disrupt the group dynamic.

## 4 Discussion

Recognizing the many benefits that group therapy can bring to people with chronic pain, we have developed an advanced EMDR group therapy program, despite the fact that EMDR has largely been researched as an individual intervention. This program aims to create a supportive and empowering environment for patients, facilitating trauma processing, the acquisition of effective pain management skills, and the formation of helpful bonds with others who share similar experiences. Early pilot studies have been encouraging but have also highlighted some unique aspects that need to be considered in implementation, including the group setting, length and breadth of sessions, content design, inclusion of additional therapeutic components, safety considerations and potential patient exclusion guidelines.

### 4.1 Format and scope of the group offer

Our intensified EMDR group therapy program for patients with chronic pain combines EMDR therapy with physical activation and education. It uses both individual and group work to help patients cope with stressful experiences and activate resources to manage their suffering. During the pilot phase, we learned that building strong group cohesion is crucial for successful EMDR group therapy. Starting EMDR work too early can be unproductive and cause patients to withdraw from therapy. To avoid this, our program emphasizes a period of intensive group work before EMDR therapy begins. This consists of setting group rules and various exercises to help patients get to know each other better. We have also found that starting with the less intensive EMDR-IGTP-OTS protocol, where the traumatic experience is drawn and symbolized, can ease patients into therapy and improve their acceptance. This approach has led to better acceptance of the more intensive G-TEP. Relative to the EMDR-IGTP-OTS protocol, the G-TEP protocol induced more emotional discomfort among the participants. Although it appeared to promote a more intensive processing, it simultaneously necessitated that the participants maintain a high level of trust in the setting. Commencing with this protocol was found to be disadvantageous, as it made certain participants uncomfortable and even resulted in some quitting the treatment. Significant improvements in exposure work were achieved by implementing a well-structured approach that included an extended group work phase at the beginning of the intervention. Targeted efforts to promote group cohesion and a gradual integration of the EMDR-IGTP-OTS protocol for exposure work effectively addressed potential challenges, ultimately leading to improved outcomes. A clearly structured and consistent treatment framework (fixed therapy times, consistent breaks, guided walking) has also proved helpful. This imitates to some extent the protocol-based approach of the standard EMDR protocol and thus offers a reliable framework for dealing with emotional stress.

### 4.2 Extension of therapy sessions

Regular group therapy sessions usually consist of weekly or fortnightly sessions of 50–90 min each (Kaptan et al., 2021). However, in the context of EMDR group therapy, this time frame has proven to be insufficient. In pilot studies, the session duration was gradually increased to 3 h and finally set at around 5 h. There were two main reasons for this adaptation: firstly, some participants needed a longer preparation time before addressing central distressing and traumatic issues, and a longer session length allowed for two processing and exposure units per session. Secondly, the standard therapy time of 50 or 90 min was not sufficient for many participants to achieve a satisfactory reduction in subjective distress. By extending the session time and incorporating repeated exposure within a session, interspersed with resource work, these difficulties were overcome, and the effectiveness and acceptance of the treatment was significantly improved.

### 4.3 Integration of complementary therapeutic elements into the EMDR treatment program

Extending the duration of individual therapy sessions is challenging because participants tend to become increasingly fatigued as the session progresses. This was due to the prolonged engagement required during therapy, which gradually drains participants’ energy levels and reduces their overall level of stamina. Participants reported severe mental exhaustion after the emotional arousal and subsequent desensitization, so that further treatment was not considered helpful by the therapists in this situation. Relaxation exercises or a “free break” also did not have a sufficient impact on the group’s perceived energy level. The integration of activating elements such as guided walking proved to be an effective solution to overcome the participants’ exhaustion caused by the long therapy sessions and to restore their receptivity. With this in mind, we included a joint activation exercise in the treatment program, where patients were invited to take a brisk walk accompanied by therapists on a voluntary basis. Furthermore, the effects of trauma confrontation were enhanced by subsequent exercise. This was well accepted by the patients and could positively influence the subsequent working atmosphere (Bryant et al., 2023).

### 4.4 Group cohesion and group therapy work

Over the course of the pilot phase, it had become apparent that a sufficient degree of trust in the therapeutic setting as well as a supporting group cohesion is an important prerequisite for working on emotionally stressful content. To actively promote this, various interventions can be carried out in small and large group work. For example, a group therapy intervention was carried out at the beginning of a treatment day, in which the participants had to choose a card from many different cards with different motives that they felt applied to them. They then share with the group why they chose that card. This exercise actively promotes group cohesion as the participants gain insights into the personality and emotional state of the other group members. In addition, this exercise can also serve as a mood screening, as it gives an overview of the group’s current mood and state of mind.

### 4.5 Safety and possible contraindications

Safety is an important aspect to consider when using exposure-based therapies. Studies on the use of EMDR in the treatment of patients with post-traumatic stress disorder have not found evidence of increased safety risks in group settings (Kaptan et al., 2021). Of the 22 studies that examined the use of EMDR in group settings, six reported no adverse effects, while five reported that individual EMDR sessions were occasionally required and some participants experienced an increase in depression, stress and anger during therapy (Kaptan et al., 2021). Overall, the available data do not suggest a specific safety risk for EMDR group therapy, but the data are not yet sufficient for a conclusive assessment.

However, the risk of possible side effects from the exposure elements is higher than in purely educational therapy groups. Therefore, it may well be that individual participants need additional support during or after the group intervention. This should be considered when planning and implementing the EMDR group intervention. On the one hand, a possibility should be created to offer individual sessions to individual participants who need additional support after the group intervention. Our experience has shown that this only concerns individual cases and that one or two sessions are usually sufficient here. However, it is much more common that individual participants need additional support during the EMDR intervention—especially in the first half of the treatment intervention this can happen—when patients on the one hand become increasingly courageous to go into exposure, but at the same time the presence of the safe “here and now” through the group and the activation of their own resources is not yet sufficiently consolidated. For this purpose, the concept of the emotional support team was introduced (Jarero et al., 2006): In addition to the group leader, a therapist (per 10 participants) should always take part in the group to be able to offer additional support to individual participants who need it during the therapy. This therapist team does not have to actively participate in the EMDR work but should be able to help individual patients regain a safe state and address additional needs.

Another risk of the group setting is that individual participants may become distressed by the description of the traumatic experiences of other members. This potentially carries the risk of a possible “re-traumatization,” at least if the affected persons again enter a state of extreme fear and helplessness that overwhelms their individual coping skills. During an exposure, it is the therapist’s responsibility to ensure that participants do not lose contact with the safe present in order to minimize the risk of losing control again. The therapist also needs to create a supportive group atmosphere that provides an appropriate level of safety, control and self-esteem for all participants.

In the pilot phase, the importance of evaluating individual suitability for group therapy EMDR was evident. The individual pre-session between the patient and therapist played a crucial role in this process. Here, the presence of possible contraindications (see Table 3) and the participant’s sufficient stability should be critically examined. In addition, the first therapist’s impression of the participant during the first stabilization exercises and exposure units in the group can play an important role: If participants have difficulty reducing stress during the stabilization exercises or early exposures offered, consideration can be given to exclude them from the group program and offering them individual treatment. While it is important to critically evaluate group suitability before beginning therapy, it should also be evaluated after the regular treatment program is completed whether individual participants need additional support. If necessary, additional (individual) sessions should be offered.

## 5 Conclusion

In summary, our experience with an intensified outpatient group treatment with EMDR suggests that we have developed a promising treatment program for patients with chronic pain. This provides new and promising treatment options, especially for those patients with pain who have high levels of emotional distress and post-traumatic stress symptoms. The opportunity to share with other patients and the pain management strategies learned are particularly promising. We hope that future studies will take up this approach to further develop and optimize the program and prove its effectiveness. This could help to better meet the needs of this special group of patients and improve their quality of life.

## Ethics statement

The study protocol was approved by the Ethics Research Committee II of the Faculty of Medicine, University of Heidelberg (S-767/2022) and will be carried out in compliance with the Helsinki Declaration. Written consent was provided from all participants before enrollment.

## Author contributions

SV: Conceptualization, Project administration, Writing – original draft, Writing – review & editing, Data curation, Formal analysis, Investigation, Methodology. AD: Conceptualization, Writing – original draft, Writing – review & editing. JR: Conceptualization, Writing – original draft. FE: Writing – review & editing. SW: Methodology, Writing – review & editing. EB: Data curation, Investigation, Methodology, Writing – review & editing. H-CF: Funding acquisition, Resources, Writing – review & editing. IJ: Supervision, Writing – review & editing. GS: Supervision, Writing – review & editing. JT: Conceptualization, Writing – original draft, Writing – review & editing, Project administration.
