# Supplementary material for: Group eye movement desensitization and reprocessing (EMDR) in chronic pain patients
Source: Front Psychol. 2024 Feb 22;15:1264807. doi: 10.3389/fpsyg.2024.1264807 (PMC10919217; doi:10.3389/fpsyg.2024.1264807)
Supplement: Supplementary file 1 [file Data_Sheet_1.pdf]

Supplemental material

Table S1: Overview of the 1st day of treatment

Table S2: Overview of the 2nd day of treatment

Table S3: Overview of the 3rd day of treatment

Table S4: Overview of the 4st day of treatment

Table S1: Overview of the 1st day of treatment

Day 1	Content
introduction of participants<br>25 min	▪ Introduction with name and age, brief mention of the pain symptoms, since when they have existed and how they affect everyday life.
Education<br>20 min	▪ What is pain memory;<br>▪ What is pain?<br>▪ How does pain develop?
Plenum and small group work<br>30 min	▪ Playful getting to know each other: line up by first letter of first name (alphabetical), distance of place of residence from clinic, distance, first letter of favourite place (alphabetical)<br>▪ Partner work: Find out what your partner needs to get better? What are my expectations of the treatment? (What is my pain history?) & report about the other person in the large group.
Break<br>15 min	
Education<br>EMDR and Pain<br>20 min	▪ Lecture "What is EMDR?: EMDR for pain, EMDR in the group?" Side effects (rather short, as discussed in preliminary interviews) Stop signs, emergencies & group rules
PT-OTS<br>70 min	▪ Target: stressful things in connection with pain, search through "mental pain film".
Walk<br>50 min<br>+ 5 min break	▪ Physical regulation and promotion of group interaction
PT-OTS<br>60 min	▪ Target: stressful things in connection with pain, search through "mental pain film".
5 min break	▪ Short physical activation (5 min)
Closing session<br>30 min	▪ "How am I doing now?" "What have I experienced today"<br>▪ Reference to post-processing, impulse for self-care
Overall duration: 5.25 h	

Legend: PT-OTS: Integrative Group Treatment Protocol Adapted for Ongoing Traumatic Stress; G-TEP: Group Traumatic Episode Protocol

Table S2: Overview of the 2nd day of treatment

Day 2	Content
Arrival<br>30 min	▪ Arrival, orientation phase,<br>▪ Short round about the previous day
Break<br>5 min	▪ Setup G-TEP
G-TEP<br>120 min	▪ Target: stressful things in connection with pain, search through "mental pain film".
Walk<br>50 min<br>+ 5 min break	▪ Physical regulation and promotion of group interaction
OTS<br>40 min	▪ Target: stressful things in connection with pain, search through "mental pain film".
Activation and break	▪ Short physical activation (10 min)<br>▪ 5 min. Break
Education<br>30 min	▪ Turn over: "What is pain?": Pain as an alarm signal, pain and memory, pain and relaxation, pain and movement
Closing session<br>10 min	▪ "How am I doing now?" "What have I experienced today"<br>▪ Reference to post-processing, impulse for self-care
Overall duration:<br>5.00 h	

PT-OTS: Integrative Group Treatment Protocol Adapted for Ongoing Traumatic Stress; G-TEP: Group Traumatic Episode Protocol

Table S3: Overview of the 3rd day of treatment

Day 3	Content
Arrival<br>30 min	▪ Choose cards with picture impulses: "How am I here today?": Choose a card and then discuss in partner work and share in total group afterwards.
Break<br>5 min	▪ Setup G-TEP
G-TEP<br>110 min	▪ Target: stressful things in connection with pain, search through "mental pain film".
Walk<br>50 min<br>+ 5 min break	▪ Physical regulation and promotion of group interaction
OTS-Pain<br>40 min	▪ Imagination exercise: (e.g. ray of light, body resource, place of well-being)<br>▪ Target: Pain picture; instruction to visualise and draw the pain sensation with a coloured pencil<br>▪ Imagination exercise (e.g. ray of light, body resource, place of well-being)
Break<br>5 min	
Education<br>30 min	▪ Pain and Stress
Closing session<br>10 min	▪ "How am I doing now?" "What have I experienced today"<br>▪ Reference to post-processing, impulse for self-care
Overall duration:<br>5.00 h	

PT-OTS: Integrative Group Treatment Protocol Adapted for Ongoing Traumatic Stress; G-TEP: Group Traumatic Episode Protocol

Table S4: Overview of the 4th day of treatment

Day 4	Content
Arrival<br>30 min	▪ Choose an animal figure: "Which figure fits today?": Choose a figure and discuss in partner work, then share as a group, "Why did I choose this animal?"
Break<br>5 min	▪ Setup G-TEP
G-TEP<br>100 min	▪ Target: stressful things in connection with pain, search through "mental pain film".
Walk<br>50 min<br>+ 5 min Break	▪ Physical regulation and promotion of group interaction
Absorption practice<br>90 min	▪ Baseline: pain, pain management skills
Individual reflection<br>5 min	▪ "What do I take with me/What do I want to leave there" write on cards
Closing session<br>20 min	▪ Joint reflection based on the cards<br>▪ Reference to post-processing over a longer period of time and possibility to contact if necessary
Overall duration:<br>5.00 h	

PT-OTS: Integrative Group Treatment Protocol Adapted for Ongoing Traumatic Stress; G-TEP: Group Traumatic Episode Protocol
